# Advancing muscle aging and sarcopenia research through spatial transcriptomics

**DOI:** 10.1016/j.afos.2025.05.002

**Published:** 2025-06-12

**Authors:** Byeong-Don Min, Chae Young Hwang, Doyeong Kim, Sang-Yun Kim, Jayasinghage Nirmani Chathurangika Jayasinghe, Minh Nhat Tran, Sang-Min Park, Ki-Sun Kwon

**Affiliations:** a College of Pharmacy, Chungnam National University, Daejeon, South Korea; b Aventi Inc, Daejeon, South Korea; c Faculty of Traditional Medicine, Hue University of Medicine and Pharmacy, Hue University, Thua Thien Hue, Viet Nam

**Keywords:** Muscle aging, Sarcopenia, Spatial transcriptomics, Muscle atrophy, Microenvironment

## Abstract

Sarcopenia, defined as a decline of muscle mass and function with aging, poses significant health challenges. This decline is driven by multiple factors including cellular dysfunction, mitochondrial impairments, oxidative stress, and chronic inflammation, all of which collectively disrupt muscle homeostasis and regeneration. Despite the lack of approved treatments for sarcopenia, the search for effective therapies continues as various pharmacological interventions are currently in the early stage of development. Recent advances in transcriptomics have enhanced our understanding of the molecular and cellular mechanisms underlying skeletal muscle aging. In particular, spatial transcriptomics (ST) has revolutionized the field of sarcopenia research by capturing the spatial context of gene expression to uncover site-specific regulation and cellular interactions within tissues. In this review, we explore the current knowledge of sarcopenia, the mechanisms underlying muscle aging, and recent developments in therapeutic strategies. Furthermore, we examine recent studies employing ST that have provided critical insights into the spatial heterogeneity of muscle aging and atrophy, revealing novel cellular and molecular targets for intervention. By connecting muscle pathophysiology with spatial information, ST holds promise for guiding the development of novel sarcopenia therapies, ultimately improving outcomes for aging populations worldwide.

## 1 Introduction

Sarcopenia, which refers to the progressive loss in skeletal muscle mass and function with age, has received significant attention in clinical and research settings [1]. This condition increases the risk of falls, frailty, weakness, and metabolic disorders, leading to higher healthcare costs and a lower quality of life [2]. As developed countries face a demographic shift toward an aging population, sarcopenia has become as an urgent public health concern linked to increased morbidity and mortality among older adults [3]. The World Health Organization (WHO) has recognized the clinical significance of sarcopenia and has officially classified sarcopenia with its own ICD-10 code, emphasizing it as a unique medical condition [4].

Clinically, sarcopenia is broadly classified into primary and secondary forms [5,6]. Primary sarcopenia refers to muscle loss driven predominantly by intrinsic aging, whereas secondary sarcopenia is induced by extrinsic factors such as inactivity (eg, disuse, denervation, or tenotomy), chronic disease (eg, organ failure, systemic inflammation including cancer-induced cachexia), malnutrition, or drug exposure. Despite their different initiating factors, both primary and secondary sarcopenia converge on shared molecular mechanisms, including cellular dysfunction, mitochondrial impairment, oxidative stress, low-grade inflammation, and epigenetic changes. However, distinct pathological trajectories can be observed between the two forms. Skeletal muscle is a heterogeneous tissue composed of multiple muscle fibers, along with satellite cells, fibroblasts, vascular endothelial cells, and immune populations that collectively maintain muscle homeostasis [7,8]. In primary sarcopenia, aging disrupts the balance between these fiber types, and the decline in muscle stem cell function, particularly in satellite cells, exacerbates muscle degeneration [[9], [10], [11]]. In contrast, secondary sarcopenia, depending on the underlying cause, often leads to more rapid and heterogeneous patterns of muscle atrophy [[12], [13], [14], [15]]. Although emerging pharmacological interventions targeting the biological pathways involved in sarcopenia are currently under development, no drugs have been officially approved to treat sarcopenia to date [16]. Therefore, additional foundational research is essential to unravel the intricate molecular mechanisms underlying this condition.

**Fig. 1:**
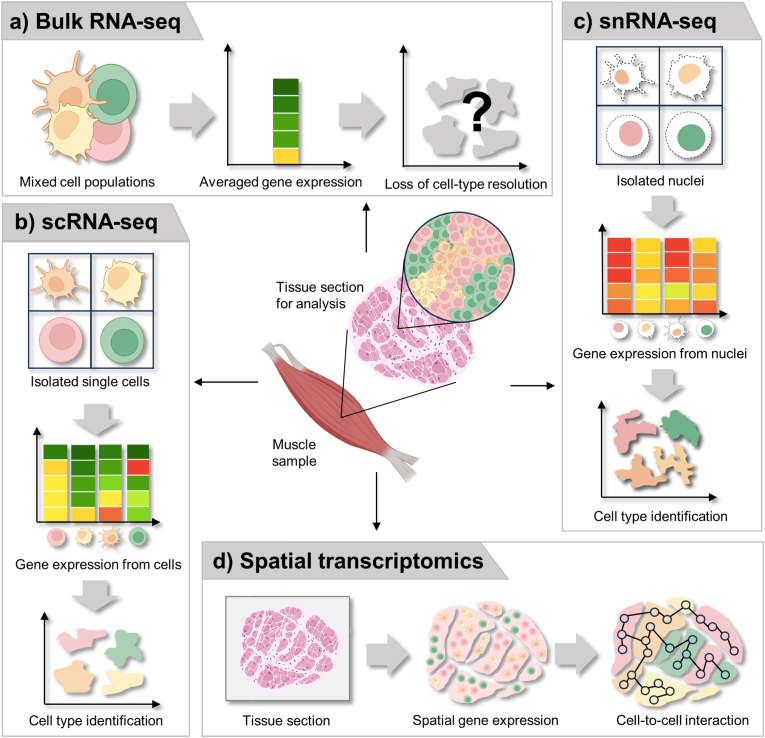
Comparative overview of transcriptomic approaches. a) Bulk RNA sequencing (bulk RNA-seq) analyzes the entire pool of RNA extracted from a muscle sample, providing an average gene expression profile across all cell populations but lacking the resolution to distinguish individual cell populations. b) Single-cell RNA sequencing (scRNA-seq) enables the identification of distinct cell populations within muscle tissue by isolating individual cells, generating cell-specific gene expression profiles, and mapping cellular diversity. c) Single-nucleus RNA sequencing (snRNA-seq) captures RNA from isolated nuclei instead of whole cells, enabling the capture of pre-mRNA and reducing bias caused by the dissociation process, offering significant advantages. d) Spatial transcriptomics (ST) integrates spatial information with gene expression data, allowing the analysis of tissue organization, cell-cell interactions, and the spatial dynamics of gene expression within tissues.

Advancements in transcriptomic technologies, including bulk RNA-seq, single-cell RNA-seq (scRNA-seq), and single-nucleus RNA-seq (snRNA-seq), have elucidated the transcriptional changes that govern biological processes [17,18]. These approaches have provided valuable insights into the global gene expression profiles of tissues and the heterogeneity of individual cells (Fig. 1). However, these conventional methods lack the spatial context required to fully capture the complexity of muscle architecture and its surrounding cellular dynamics. Recently, spatial transcriptomics (ST) has emerged as an innovative approach to overcome this limitation by capturing gene expression data linked to spatial information [19]. ST maps transcripts in situ, offering the precise location of gene expression within tissues. This capability promises to significantly advance sarcopenia research by enabling the examination of previously inaccessible aspects of muscle tissue organization and microenvironmental interactions.

This review examines the cellular and molecular foundations of sarcopenia, with a focus on the mechanisms driving muscle aging (Section 2). It next explores the emerging therapeutic strategies, including pharmacological interventions and natural products, aimed at mitigating sarcopenia (Section 3). Lastly, it highlights the potential of ST to advance sarcopenia research by unraveling the spatial organization of the muscle microenvironment and its contribution to muscle degeneration and regeneration (Section 4). Although this review primarily focuses on age-related primary sarcopenia, it also incorporates mechanistic insights from secondary sarcopenia models and other myopathies where ST data are relatively more abundant, thereby providing a broader framework for understanding and targeting sarcopenia-related muscle pathology.

## 2 Mechanisms underlying muscle aging and sarcopenia

### 2.1 Changes in muscle fiber types

Skeletal muscle consists of two main fiber types, Type I (slow-twitch) and Type II (fast-twitch), each with distinct metabolic and functional properties [7,8]. Type I fibers, also known as slow oxidative fibers, are characterized by slow contraction speeds, high resistance to fatigue, and reliance on aerobic metabolism for energy production [20]. Because they contain a large number of mitochondria, abundant myoglobin, and a dense network of capillaries, they have a dark red appearance and high endurance capacity. As a result, Type I fibers are primarily employed in activities requiring sustained, low-intensity contractions, such as maintaining posture or running long distances.

In contrast, Type II fibers contract more rapidly and generate greater force than Type I fibers, but they fatigue more readily. They are further subdivided into Type II_A_ (fast oxidative) and Type II_X_ (fast glycolytic, previously known as Type II_B_ in humans). Type IIX fibers have the fastest contraction speed and highest force production among all fiber types [7,8]. However, they fatigue rapidly owing to their reliance on anaerobic glycolysis for ATP generation [20]. These fibers contain fewer mitochondria and less myoglobin compared to those in other fibers, resulting in a paler appearance. Consequently, Type II_X_ fibers are primarily recruited for short-duration, high-intensity activities, such as sprinting or weightlifting. Meanwhile, Type II_A_ fibers utilize both aerobic and anaerobic metabolism, allowing them to generate moderate force while demonstrating reasonable resistance to fatigue [20]. These intermediate characteristics between Type I and Type II_X_ fibers make them suitable for activities requiring a balance of strength and endurance.

Aging is closely associated with a preferential loss of Type II muscle fibers, as supported by multiple studies [[9], [10], [11]]. This selective loss results in decreased muscle power, a reduced capacity for rapid movements, and an increased risk of falls among older adults [11]. The mechanisms underlying this phenomenon include age-related denervation, subsequent reinnervation by slow motor neurons, heightened susceptibility to oxidative stress, and a decline in anabolic processes [10]. These age-associated changes in muscle fiber composition emphasized the need for targeted interventions to mitigate their impact on aging populations [9,10].

The age-related loss of Type II muscle fibers is partially mitigated by compensatory adaptations in Type I fibers, which begin to express genes characteristic of Type II subtypes, including *MYH4* (MyHC IIb), *MYH1* (MyHC IIx), and *MYH2* (MyHC IIa) [7]. Additionally, recent snRNA sequencing data reveal that aged slow-twitch myofibers upregulate fast-type markers (eg, *MYH1*, *MYH2*) to compensate for the loss of Type II fibers [21]. Moreover, regeneration of remaining Type II fibers is enhanced in some aged individuals, attributed to improved satellite cell responsiveness associated with increased capillarization [22]. During motor unit remodeling, loss of Type II motor neurons leads to the reinnervation of some muscle fibers by Type I motor neurons, inducing changes in fiber type composition [9,10]. Strength-trained master athletes, for example, exhibit better preservation of Type II fibers compared to endurance-trained or recreationally active older adults [11]. However, direct evidence of Type I fibers expressing genes characteristic of Type II subtypes remains limited. These compensatory mechanisms likely represent adaptive responses that help to partially offset the decline in muscle function associated with aging.

In contrast to primary sarcopenia, secondary sarcopenia exhibits a distinct pattern of muscle fiber atrophy and is driven by different underlying mechanisms. In particular, extrinsic factors such as prolonged disuse, chronic systemic disease, or malnutrition often result in a disproportionately greater atrophy of Type I fibers compared to that seen in normal aging [23]. This fiber-type–specific vulnerability is especially evident under conditions such as extended bed rest, limb immobilization, or cachexia, which impose sustained inactivity and catabolic stress [24,25]. Because Type I fibers are essential for fatigue-resistant contractions and postural support, their preferential degeneration in secondary sarcopenia leads to functional impairments including reduced endurance, increased fatigability, and compromised postural control.

### 2.2 Cellular dysfunction

Satellite cells, the primary stem cells responsible for skeletal muscle regeneration, play a vital role in maintaining muscle mass. However, with aging, both the number and function of satellite cells decline, significantly impairing the ability of muscles to repair and regenerate [26]. This dysfunction is partly attributed to oxidative stress and changes in systemic factors and environmental cues that regulate satellite cell activity. A study demonstrated that age-related alterations in the muscle stem cell niche contribute to a decline in satellite cell function, underscoring the critical role of the cellular microenvironment in muscle aging [27]. In particular, elevated levels of transforming growth factor-β (TGF-β) activate Smad3 signaling and upregulate cell cycle inhibitors such as *p16INK4A*, thereby promoting satellite cell senescence and impairing proliferation [28]. Additionally, reduced levels of growth differentiation factor 11 (GDF11) in the circulation diminish the regenerative capacity of satellite cells [29]. Moreover, age-related extracellular matrix (ECM) fibrosis coupled with elevated oxidative stress impairs integrin-mediated signaling within the niche, contributing to functional decline [30]. However, previous studies have suggested that satellite cells may be dispensable for the maintenance of muscle mass during normal aging. In particular, genetic ablation of satellite cells in sedentary adult mice did not exacerbate age-related muscle atrophy or reduce fiber cross-sectional area [31,32]. These findings suggest that although satellite cell dysfunction compromises regenerative capacity, it is unlikely to be the primary driver of muscle loss in primary sarcopenia. However, given the essential role of satellite cells in injury-induced regeneration, their function may be more relevant in the context of secondary sarcopenia, where repeated damage or systemic disease places greater demands on muscle repair mechanisms.

Mitochondrial dysfunction is another prominent factor in sarcopenia development. Age-mitochondria showed key characteristics including the decreased oxidative phosphorylation (OXPHOS) capacity, accumulation of mitochondrial DNA (mtDNA) mutations and deletions, increased production of reactive oxygen species (ROS), and reduced efficiency of mitochondrial DNA repair mechanisms, particularly base excision repair [33,34]. Aging is associated with reduced expression of mitochondrial OXPHOS genes and proteins, leading to diminished ATP production capacity [35]. In aged skeletal muscle, significant decreases in the expression and enzymatic activity of OXPHOS complexes I, II, IV, and V have been observed [36]. This is accompanied by diminished levels and activity of PGC-1α, a master regulator of mitochondrial biogenesis, which exacerbates mitochondrial dysfunction [34,37,38]. Collectively, these age-related impairments disrupt cellular energy metabolism and are considered key drivers of sarcopenia pathogenesis. Furthermore, accumulation of mtDNA mutations leads to mitochondrial dysfunction and muscle wasting in aged mice [39].

Disruption of protein homeostasis (proteostasis) also plays a significant role in muscle aging. Aging skeletal muscle exhibits anabolic resistance, characterized by impaired mTORC1 signaling activation and diminished ribosome biogenesis due to reduced ribosomal DNA transcription and ribosomal protein synthesis, along with elevated AMP-activated protein kinase (AMPK)-mediated catabolic signaling [40,41]. Transcriptional dysregulation of the ubiquitin–proteasome system has also been reported. Pro-inflammatory cytokines upregulate 20S proteasome subunits, while age-related declines are observed in proteasome activators such as *PSMC4* [[42], [43], [44]]. In addition, stress-induced E3 ligases, including MuRF1 and Atrogin-1, show attenuated expression in aged muscle [45]. Impaired autophagy further contributes to proteostasis failure. Reductions in *ATG7* expression and dysfunction of Parkin- and Mitofusin 2 (Mfn2)-mediated mitophagy compromise mitochondrial quality control, thereby exacerbating mitochondrial dysfunction and accelerating sarcopenia progression [34,[46], [47], [48]].

### 2.3 Molecular and epigenetic changes

Sarcopenia is driven by several interconnected molecular mechanisms. One of the most studied pathways is the insulin-like growth factor (IGF)-1/phosphoinositide 3-kinase (PI3K)/Akt/mammalian target of rapamycin (mTOR) signaling cascade, which shows reduced activity in aged muscle, impairing protein synthesis and muscle growth [49,50]. Conversely, myostatin, a negative regulator of muscle growth, is upregulated, further contributing to muscle atrophy. Increased oxidative stress is another major factor, leading to the accumulation of damage to cellular components, mitochondrial dysfunction, and impaired satellite cell function [51,52]. This oxidative damage initiates a cascade of deleterious effects, including activation of apoptotic pathways and chronic tissue degeneration.

Chronic low-grade inflammation, often referred to as "inflamm-aging," plays a significant role in sarcopenia progression [53]. This process is characterized by elevated levels of pro-inflammatory cytokines, such as interleukin (IL)-6 and tumor necrosis factor (TNF)-α, which activate catabolic pathways in muscle fibers and disrupt the balance between muscle and immune system function [54]. In addition, higher levels of inflammatory markers were associated with accelerated muscle strength decline, suggesting their role as both biomarkers and potential therapeutic targets [55].

Additionally, age-related alterations in the epigenetic landscape, particularly DNA methylation, profoundly impact muscle-specific gene expression and contribute to sarcopenia. For example, a study identified extensive changes in methylation patterns linked to sarcopenia-related phenotypes, such as reduced grip strength and lean mass, suggesting a potential role for epigenetic therapies in reversing these alterations [56].

In summary, sarcopenia results from the interplay of muscle fiber composition changes, cellular dysfunction, and molecular alterations. Aging leads to a preferential loss of Type II fibers, satellite cell dysfunction, mitochondrial impairments, and disrupted protein homeostasis, all contributing to reduced muscle regeneration and strength. Additionally, molecular mechanisms such as oxidative stress, chronic inflammation, and epigenetic modifications exacerbate muscle atrophy. Although these processes are interconnected and complex, ST offers a unique ability to map gene expression within the muscle microenvironment, enabling a deeper understanding of tissue-specific interactions and guiding the development of novel interventions for sarcopenia.

## 3 Emerging treatment strategies for sarcopenia

### 3.1 Current interventions and limitations in sarcopenia therapeutics

Sarcopenia affects approximately 20% of people over 60 years of age, impacting more than 150 million individuals worldwide [57]. Despite its prevalence and impact, there are currently no approved drug treatments for sarcopenia [16]. The mainstay of sarcopenia management currently revolves around lifestyle interventions, primarily comprising exercise and nutritional support. Resistance training has shown efficacy in improving muscle strength and function [58], while adequate protein intake is crucial for maintaining muscle mass. However, these interventions may not be sufficient or feasible for all patients, particularly frail older individuals with multiple comorbidities [57].

Recent research efforts are focusing on innovative pharmacological treatments targeting the biological pathways underlying sarcopenia. Emerging pharmacological interventions for sarcopenia are showing promise alongside traditional exercise and nutritional approaches [59,60]. Furthermore, therapeutic strategies utilizing natural products are also being explored, as they hold potential to modulate key pathways related to muscle health [[61], [62], [63]]. These interventions target various pathways involved in muscle growth, inflammation, and energy metabolism. Although no drugs have been approved specifically for sarcopenia treatment by regulatory agencies such as the Food and Drug Administration (FDA) and European Medicines Agency (EMA), several promising candidates are in various stages of clinical development (Table 1).

**Table 1:** Pipeline overview of candidate drugs for sarcopenia treatment.

Development stage	Company	Drug
Phase 2	Biophytis	Sarconeos (BIO101)
Phase 2	MyMD Pharmaceuticals	Isomyosamine (MYMD-1)
Phase 2	Stealth BioTherapeutics	Elamipretide
Phase 2	Novartis	Bimagrumab (BYM338)
Phase 2	Eli Lilly	LY2495655
Phase 2	Regeneron	Trevogrumab (REGN1033)
Phase 2b	Veru Healthcare	Enobosarm (GTx-024)
Phase 2	Viking Therapeutics	VK5211
Phase 2a	GTx	MK-0773
Phase 2	Cytokinetics	Reldesemtiv (CK-2127107)
Phase 2a	Aventi	AVTR101
Phase 1	NMD Pharma	NMD670
Non-clinical	MitoRx Therapeutics	AP39
Non-clinical	Turn Biotechnologies	TRN-005
Non-clinical	AAVogen Inc.	AVGN7
Non-clinical	Neurotune AG	NT-1654
Research	Milo Biotechnology	AAV1-follistatin

### 3.2 Progress and prospects in sarcopenia therapeutics

Myostatin inhibitors have demonstrated potential in increasing muscle mass and strength [[64], [65], [66]]. Bimagrumab, a monoclonal antibody developed by Novartis, which targets activin Type II receptors, has shown promise in treating sarcopenia in older adults. Clinical trials have demonstrated its ability to significantly increase muscle mass and reduce fat mass in patients with sarcopenia [65,67]. However, its impact on functional outcomes has been mixed, with some studies showing limited improvements in muscle strength and physical performance. Currently, the investigation of bimagrumab in combination with anti-obesity medications is in the clinical trial phase.

Selective androgen receptor modulators (SARMs) have shown improvements in lean body mass and physical function [68,69]. Enobosarm, a nonsteroidal SARM, demonstrates high affinity for androgen receptors and exhibits tissue-selective effects, promoting muscle and bone growth while minimizing effects on other tissues. Clinical studies, including two phase 3 trials (POWER 1 and POWER 2), have shown improvements in lean body mass and physical function, especially in older populations and patients with cancer-related cachexia [70]. Preliminary data from these trials have indicated the potential of enobosarm in preventing muscle wasting in patients with advanced non-small cell lung cancer undergoing chemotherapy, but complete results are pending.

Mitochondria-targeted interventions have emerged as a promising approach to develop treatments for age-related sarcopenia [71]. Elamipretide, a mitochondria-targeted peptide, demonstrates high affinity for cardiolipin in the inner mitochondrial membrane and exhibits tissue-protective effects, promoting mitochondrial function and reducing oxidative stress [[72], [73], [74]]. Clinical studies, including phase 1 and 2 trials, have shown improvements in mitochondrial respiration and ATP production, especially in patients with mitochondrial dysfunction and age-related disorders, such as primary mitochondrial myopathy and intermediate age-related macular degeneration [[75], [76], [77]]. However, recent findings from a phase 3 randomized clinical trial assessing the efficacy and safety of elamipretide in patients with primary mitochondrial myopathy showed no significant improvement compared to placebo at 24 weeks [78]. Despite these results, preliminary data from this trial suggests that elamipretide may have potential in addressing various age-related conditions. Research is ongoing to explore the applications of elamipretide in neurodegenerative diseases and other mitochondrial-related disorders [79,80].

Natural compounds are also being explored as potential interventions for age-related sarcopenia [81]. Sarconeos (BIO101), targeting the MAS receptor, has shown promise in preclinical studies and early clinical trials [82]. By activating the MAS receptor, Sarconeos enhances muscle function through multiple pathways, including the PI3K/Akt/mTOR and AMPK/acetyl-CoA carboxylase (ACC) signaling cascades [83]. This mechanism enhances muscle function by promoting mitochondrial activity and reducing muscle loss. Clinical studies, including the SARA-INT phase 2 trial, have demonstrated improvements in physical performance, particularly in the 400-m walk test, a key measure of mobility in individuals with sarcopenia (https://www.biophytis.com). Biophytis is currently has FDA approval to conduct a phase 3 study (SARA-31) on Ruvembri™ (20-hydroxyecdysone) in patients with sarcopenia.

Isomyosamine, (MYMD-1), a synthetic alkaloid derived from the tobacco plant, inhibits the production and release of cytokines in serum, including interferon (IFN)-γ, IL-2, IL-10, and TNF-α, in a dose-dependent manner [84]. Leveraging this mechanism, MyMD Pharmaceuticals is developing MYMD-1, as an oral treatment for sarcopenia and other inflammatory conditions. MYMD-1 has demonstrated potential in reducing inflammatory biomarkers in older patients with sarcopenia, frailty, and other inflammatory conditions (MyMD Pharmaceuticals, 2023). Currently, the company is preparing for phase 3 clinical trials to further evaluate the efficacy and safety of MYMD-1 in patients with sarcopenia.

AVTR101, a drug with alverine citrate as the main active ingredient, is being developed by Aventi as a treatment for sarcopenia through a mechanism that promotes muscle cell differentiation and fusion. Oral administration of alverine citrate has improved muscle mass and physical performance in aged mice [85]. Additionally, alverine citrate has also been found to enhance neuromuscular junction (NMJ) activation. This effect on NMJ activity further supports its potential as a comprehensive treatment for sarcopenia, addressing both muscle cell function and neuron-to-muscle communication. Currently, Aventi is conducting a Phase 2a clinical trial to evaluate the efficacy and safety of AVTR101 in patients with sarcopenia.

These emerging therapeutic candidates highlight the dynamic and evolving landscape of pharmacological interventions for sarcopenia. While the primary therapeutic focus of emerging pharmacological interventions is age-related sarcopenia, several candidates also exhibit potential applicability to secondary sarcopenia contexts, including cancer cachexia, thereby indicating a broader therapeutic scope for future clinical translation.

## 4 Spatial transcriptomics for transforming sarcopenia research

### 4.1 Advancements in spatial transcriptomics technology

Although significant progress has been made in understanding sarcopenia and developing potential treatments, many challenges remain owing to the complexity of muscle aging and its interplay with the surrounding microenvironment. Recent innovations in ST can offer groundbreaking solutions in sarcopenia research by preserving the spatial location of cells within tissues while profiling gene expression data [86]. For muscle tissues, ST provides a unique opportunity to investigate the spatial dynamics of muscle fibers, stem cells, and immune populations, offering insights into tissue remodeling and degeneration in aging and disease. Several studies have demonstrated its applications in uncovering the complexities of muscle microenvironments (Table 2).

**Table 2:** Studies and available data utilizing ST in muscle research.

Disease category	Species	Muscle site	Experimental conditions	ST platform	GEO accession code	Reference
*P*	*Homo sapiens*	Vastus lateralis	Old patient with osteoporosis and sarcopenia and young patient with muscle pain after physical exercise (N = 1, each).	Xenium	–	(108)
*S*	*Mus musculus*	Soleus	Control (N = 5) and mice underwent denervation via sciatic nerve transection, with recovery groups at 5 days (N = 2) and 7 days (N = 4).	Seq-Scope	–	(109)
*S*	*Mus musculus*	Tibialis anterior	Control (N = 8) and mice underwent denervation by crushing the sciatic nerve (N = 4).	Visium	GSE198596	(110)
*S*	*Oryctolagus cuniculus*	Supraspinatus	Healthy tissue (N = 1) compared to 2 and 16 weeks post tenotomy (N = 1). Freshly snap frozen tissues compared to tissue stored for <6 year (N = 1).	Visium	GSE210773	(111)
*O*	*Mus musculus*	Gastrocnemius	Control (N = 3) and severely dystrophic mice (N = 5).	Visium	GSE225766	(112)
*O*	*Mus musculus*	Tibialis anterior and gastrocnemius	Control (N = 3) and dystrophic mice subjected to muscle injury by cardiotoxin (N = 9).	Visium	GSE161467 GSE223813	(113)
*O*	*Mus musculus*	Gastrocnemius	Control (N = 5) and dystrophic mice (N = 7).	Visium	GSE225593 GSE226173	(114)
*O*	*Homo sapiens*	Quadriceps	Patients with sarcoidosis (N = 2).	Visium	GSE243291	(115)

P, Primary sarcopenia; S, Secondary sarcopenia; O, Other myopathy; GEO, gene expression omnibus; ST, spatial transcriptomics.

**Fig. 2:**
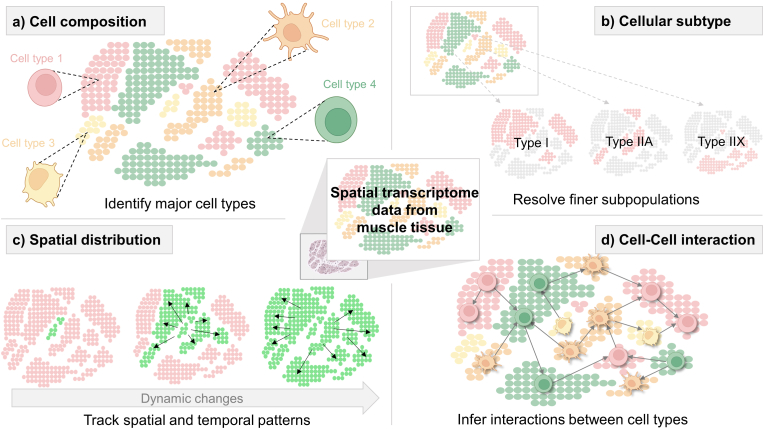
Applications of ST in sarcopenia research. a) Visualization of the diverse cell types within muscle tissue to uncover the cellular landscape. b) Identification of distinct cellular subtypes and their spatial distributions in muscle tissue. c) Dynamic changes in the spatial organization of cells within muscle tissue, demonstrating transitions and interactions over time or under different conditions. d) Cellular interactions within the muscle microenvironment, emphasizing communication between different cell types.

Various analytical techniques have been developed specifically for ST data, enabling detailed characterization of cellular composition within tissues, identification of novel cell subtypes, comprehensive analysis of spatial distributions, and advanced investigations into cell-cell communication (Fig. 2). These advancements have established ST as a widely used approach in disease studies, integrating high-resolution imaging with molecular biological techniques to investigate gene expression patterns, spatial cell distribution, microenvironmental interactions, and lesion localization, providing detailed insights into complex tissue architecture [87]. The precise spatial information in ST data enables analysis of cell-cell interactions, spatial correlations among various cell types, and functional cellular clusters within tissues [88,89]. A variety of tools have been developed and applied to investigate cell-cell interactions through the co-expression analysis of ligand-receptor interaction [89,90]. For example, CellNeighborEX is one of cutting-edge tool designed to identify potential genes involved in cell-cell interactions, surpassing the constraints of traditional ligand-receptor co-expression analysis [91]. Further, ST can advance precision medicine by tailoring therapeutic strategies to the unique tissue characteristics of each patient, thereby increasing treatment efficacy and minimizing adverse effects [87,92]. These innovations have opened promises to investigate how tissue architecture and local microenvironments shape cellular behavior in both aging and disease.

Two primary strategies are commonly used in ST. The first is image-based ST, which employs microscopy to directly visualize mRNA in tissue sections [93]. This includes techniques like Multiplexed Error-Robust Fluorescence in situ Hybridization (MERFISH) [94] and sequential Fluorescence In Situ Hybridization (seqFISH) [95], which focus on acquiring gene expression data within the context of tissue structure using microscopic imaging. These methods enable precise determination of both the location and expression level of specific mRNA molecules. MERFISH pinpoints the precise location of gene expression by assigning error-resistant fluorescent barcodes to each gene, enabling large-scale profiling, while seqFISH quantifies gene expression by sequentially recording fluorescence signals for multiple genes. Although these methods yield detailed spatial data for inter- and intracellular interactions, they are limited by the number of fluorescent signals that can be distinguished in a single experiment. MERFISH enables spatial profiling of up to approximately 1000 genes in a single experiment by using combinatorial labeling and error-robust barcoding strategies [96]. In comparison, the original implementation of seqFISH allowed spatial transcriptomic profiling of up to ∼200 genes through sequential rounds of hybridization and imaging [97,98]. Further advancements led to seqFISH+, which significantly expanded the multiplexing capacity, enabling detection of up to ∼10,000 genes in a single cell with optimized probe design and imaging cycles [95]. Consequently, the scope of genes that can be examined simultaneously is restricted, potentially constraining the study of intricate tissue interactions [99,100]. Xenium integrates automated imaging and analytical workflows, enabling the simultaneous profiling of more than 400 RNA targets within a single experiment [101]. This capability facilitates precise tracking of cell-cell interactions and detailed characterization of the tissue microenvironment, although it still falls short of whole-transcriptome coverage. Nevertheless, its scalability and user-friendly design position Xenium as a powerful platform for advancing large-scale ST research.

The second approach is sequencing-based ST, including Visium [102] and Slide-seq [103], which use spatial barcode beads to directly sequence mRNA at specific spatial locations within the tissue to generate gene expression data [104]. Visium is a method for rapidly sequencing mRNA at discrete spatial locations by affixing tissue samples to a slide and applying spatially barcoded oligonucleotides to defined regions. Slide-seq achieves higher spatial resolution by transferring tissue sections onto a slide overlaid with spatially barcoded beads and subsequently sequencing gene expression at each bead location. Unlike image-based approaches, sequencing-based approaches enable the analysis of comprehensive gene expression over a relatively large spatial range [105,106]. However, the spatial resolution is relatively low and the location of the barcode can combine transcripts from multiple cells or cell regions, which can complicate the detection of gene expression changes caused by the cellular microenvironment. Recent progress with Seq-Scope has helped mitigate these limitations by precisely identifying individual cell locations within tissues and performing high-resolution mRNA sequencing at targeted sites [107].

### 4.2 Spatial insights in primary sarcopenia

ST studies focused on genuinely age-related primary sarcopenia are still rare. However, a recent Xenium-based analysis of vastus lateralis muscle samples from an 85-year-old woman with clinically diagnosed sarcopenia and a 19-year-old control offers a detailed blueprint of what ST can reveal beyond classical bulk or single-cell datasets [108]. By manually segmenting over 110 myofibers per section and mapping 377 targeted transcripts at subcellular (≤1 μm) resolution, the authors uncovered four key spatial features of aging skeletal muscle: (i) subcellular transcript gradients, (ii) fiber-type-specific aging signatures, (iii) perimysium versus myofiber niche divergence, and (iv) age-specific rewiring of apoptotic and ECM programs.

Firstly, ST revealed a markedly higher global mRNA density in the nuclear and perinuclear regions compared to the cytoplasm in both young and old samples. This enrichment near the myonuclei provides an explanation for why aged myofibers, which contain fewer or dysfunctional nuclei, exhibit reduced overall transcriptional output, a phenomenon that would be masked in conventional bulk sequencing. In addition, ST analysis demonstrated that this nuclear and perinuclear accumulation was significantly less pronounced in Type II fibers than in Type I fibers, suggesting that fast-twitch fibers are more vulnerable to transcriptional decline with aging. These fine-scale intracellular gradients, spanning the nucleus, perinucleus, and cytoplasm, are inaccessible to traditional RNA-seq methods and emphasize the unique resolution advantage offered by ST.

Secondly, co-registration of Xenium transcript maps with serially immunostained sections for MyHC isoforms enabled precise fiber-type identification within the sarcopenic muscle. This approach revealed that Type II fibers exhibited a marked reduction in oxidative-metabolism transcripts such as *S100A1*, *PROX1*, and *HMGCS2*, while largely retaining glycolytic markers. In contrast, Type I fibers upregulated stress-response and regenerative genes including *LGR5* and *IGF1*, suggesting a compensatory anabolic response. Differential expression analysis identified 26 genes with fiber-type–specific spatial changes, including the upregulation *PROX1* and downregulation of *PVALB*, highlighting that transcriptional aging is highly localized within individual fiber types.

In addition to these transcriptional differences, histological and transcriptomic analyses together confirmed that Type II fibers were disproportionately affected by aging-related degeneration. In the sarcopenic sample, these fibers showed clear reductions in cross-sectional area and greater size variability, consistent with their increased vulnerability to atrophy. Spatial profiling further revealed upregulation of *DST*, *MEF2C*, *GATM*, *PMP22*, and *PLIN4* within Type II fibers, implicating processes such as cytoskeletal remodeling, impaired regeneration, apoptosis, and metabolic stress. Interestingly, PMP22 was also upregulated in Type I fibers, indicating a broader role in structural adaptation across fiber types. These findings reinforce that selective degeneration of Type II fibers plays a central role in the muscle weakness and functional decline observed in primary sarcopenia.

Thirdly, ST allowed for the spatial separation of contractile fibers and the fibrovascular perimysium within the same section. This revealed 191 genes differentially expressed between the two compartments. Perimysial regions showed enrichment for endothelial and ECM-associated genes such as *CAVIN1*, *VWF*, *PECAM1*, and collagen-related transcripts, mapping a micro-angiogenic and ECM-remodeling program that becomes more pronounced with aging. Such niche-specific transcript profiles would have been masked in dissociation-based methods, where fibroblast and endothelial signals dilute muscle-specific signals.

Finally, comparative analysis of young and old Type I fibers demonstrated downregulation of prosurvival *IGF1* and upregulation of proapoptotic *FAS* and ECM-remodeling genes including *COL5A2* and *TIMP4*. These changes were spatially confined to slow-twitch regions, revealing that molecular aging proceeds in a heterogeneous, patchwork manner even within the same fiber type and anatomical region.

Together, these findings illustrate what only ST can uncover: nucleus-centered transcriptional decline, fiber-type- and niche-specific gene rewiring, and highly localized degeneration patterns. Importantly, this approach has identified concrete therapeutic candidates that would likely be overlooked by conventional methods. For instance, the upregulation of *IGF1* in Type I fibers suggests a fiber-specific anabolic target, while *CAVIN1* and *PECAM1* enrichment in the perimysium points to niche-specific angiogenic remodeling as an intervention point. Additionally, stress-associated genes such as *DST*, *MEF2C*, and *PMP22*, identified predominantly in Type II fibers, offer insights into cytoskeletal stabilization and apoptotic regulation pathways. These spatially resolved targets provide new opportunities for precision strategies aimed at restoring muscle function and counteracting sarcopenia progression.

### 4.3 Lessons from spatial insights in secondary sarcopenia

While ST studies on primary sarcopenia remain scarce, multiple ST studies have examined secondary sarcopenia using *in vivo* models, including denervation and tenotomy. These models, although varied in their initiating factors, collectively provide important lessons on how extrinsic stressors reshape muscle architecture and transcriptional landscapes.

In a study of mice sampled at 3 and 7 days after denervation, ST revealed spatial patterns of damage marker expression [109]. Notably, *Xirp1*, *Enah*, and *Flnc* were initially localized around the NMJ and later spread to nearby myofibers, providing spatially resolved early markers of muscle injury that would be difficult to capture by conventional methods. ST analysis also identified novel muscle type-specific genes including *Cryab*, *Hspb1*, *Atp2a2* for Type I fibers; *Tpm1*, *Tnnt3* for Type IIa fibers; and *Mt1*, *Mt2*, *Aldoa* for Type IIx fibers, illustrating fiber-type-specific stress adaptations following denervation. Interestingly, hybrid fibers exhibiting gene expression characteristics of multiple myofiber types were also observed, suggesting a more complex transcriptional landscape. Moreover, non-muscle cells, such as macrophages and fibroblasts, were found to play key roles in ECM remodeling and tissue repair at injury sites. Terminal Schwann cells were also shown to actively contribute to ECM remodeling around the NMJ.

Another study induced nerve injury in mice and analyzed ST at early (3 days) and late (30 days) phases to investigate the processes of muscle degeneration and regeneration [110]. This study identified eight functional clusters corresponding to distinct muscle structures, including the glycolytic cortex, oxidative core, transition/mixed zones, muscular matrix scaffold, epimysium, peripheral nerves, neuromuscular junctions, and blood vessels. After denervation, glycolytic fibers exhibited higher susceptibility to atrophy, characterized by increased expression of atrophy-related factors such as Atrogin-1 and MuRF1. In addition, ST localized the polyamine imbalance specifically to glycolytic cortex, as key enzymes in the polyamine pathway were found to be innervation-dependent and predominantly concentrated in this region. Denervation led to this spatially confined metabolic vulnerability and subsequent putrescine accumulation, which further exacerbated muscle atrophy and impaired regeneration.

In a rabbit model of tendon tenotomy, ST analysis revealed distinct spatial gene expression patterns across regenerative and degenerative regions [111]. Regenerative areas exhibited high expression of genes such as *MYH8*, *MYL4*, and *ACTN3*, which are associated with muscle fiber regeneration and repair. Conversely, degenerative regions exhibited high expression of fibrosis-related genes, including *COL1A1* and *FN1*, identifying fibrosis initiation zones. This indicates active ECM remodeling and tissue fibrosis, both of which contribute to muscle deterioration. This study also demonstrated that archived tissue samples stored for over six years maintained sufficient RNA integrity, validating their suitability for ST analysis and opening avenues for retrospective investigations.

### 4.4 Lessons from spatial insights in other myopathies

While the studies discussed in the previous sections have primarily focused on primary or secondary sarcopenia, valuable insights can also be gained from ST analyses of other muscle diseases with overlapping pathological features. Among various muscle disorders, Duchenne muscular dystrophy (DMD), a severe X-linked genetic disease characterized by progressive muscle fiber degeneration, and muscular sarcoidosis, an inflammatory disease marked by granulomatous lesions in skeletal muscle, have been explored using ST approaches. Although their underlying etiologies differ, both conditions exhibit key pathological processes commonly seen in sarcopenia, including chronic inflammation, fibrosis, and impaired muscle regeneration. ST analyses of these disease models have uncovered spatially confined patterns of immune-fibrotic remodeling and disruptions in fiber–niche architecture, alongside the activation of immune and stromal circuits that similarly arise in aged muscle.

Several ST studies in DMD mouse models have provided detailed insights into the immune and fibrotic remodeling underlying muscle degeneration and regeneration. One study integrating ST with scRNA-seq constructed a high-resolution spatial atlas, revealing distinct clusters of immune and fibrotic cells expanding from injury sites, with early regenerating regions enriched for inflammatory signals such as *Ccl2*, *Ccl7*, and *Spp1* that promoted both inflammation and fibrosis [112]. Another investigation discovered a novel architectural feature, termed multilayered regenerative inflammation zones, where pro-inflammatory macrophages localized centrally, while *GPNMB*-expressing macrophages surrounded regenerating fibers and were critical for effective tissue repair; disruption of this organized structure by prednisolone impaired regeneration [113]. Additionally, a separate study identified six distinct macrophage clusters through integrated scRNA-seq and ST analysis, highlighting a Galectin-3–positive subset that drove fibrosis by promoting the differentiation of fibroadipogenic progenitors into myofibroblasts via osteopontin-mediated signaling [114]. These spatial insights redefine our understanding of dystrophic muscle pathology and offer important translational relevance to sarcopenia, where similar immune-stromal remodeling processes contribute to muscle degeneration.

In addition to dystrophic muscle conditions, a recent study applied ST to map transcriptomic changes in the muscles of patients with muscular sarcoidosis, an inflammatory disease characterized by granulomatous lesions in skeletal muscle [115]. Spatial analysis identified five distinct clusters, including granuloma, perigranuloma, and three muscle zones (proximal, intermediate, distal). Specifically, the granuloma region showed increased expression of inflammatory and fibrotic cytokines, including TNF-α, TGF-β, interferon-γ, IL-1, and IL-6, and these fibrotic signaling gradually spread to the surrounding muscle, resulting in atrophy and fibrosis. Furthermore, the proximal muscle zone showed significant downregulation of muscle-specific genes and upregulation of fibrosis-associated pathways, including epithelial-to-mesenchymal transition and fibroblast proliferation. These spatial findings reveal how inflammation and fibrosis propagate and remodel muscle tissue in muscular sarcoidosis, providing valuable parallels to the mechanisms underlying age-related sarcopenia and informing the development of targeted therapeutic strategies.

## 5 Conclusions

Sarcopenia is a complex condition influenced by multiple interconnected factors, including cellular dysfunction, oxidative stress, and chronic inflammation. While developing pharmacological interventions has shown promise, addressing sarcopenia still requires mechanistic innovation and more precise therapeutic strategies. The advent of ST represents a significant advancement for sarcopenia research. By preserving the spatial context of tissues while simultaneously capturing gene expression data, ST enables a comprehensive understanding of the interplay between muscle fibers, stem cells, and immune cells within the aging muscle microenvironment.

ST-based analyses have revealed fiber-type–specific dysregulation and niche-level remodeling that are difficult to detect using conventional bulk or single-cell approaches. For example, in primary sarcopenia, ST detects *IGF1* downregulation in Type I fibers together with *DST*, *GATM*, and *PLIN4* upregulation in Type II fibers, indicating parallel anabolic failure in slow fibers and stress-induced cytoskeletal and metabolic remodeling in fast fibers. Perimysial niches simultaneously display localized angiogenic signals, suggesting complementary perfusion-enhancing targets. In secondary sarcopenia models, ST revealed early atrophy markers such as *Xirp1*, *Cryab*, and polyamine-pathway enzymes selectively enriched in glycolytic regions, suggesting spatially restricted intervention points. In other myopathies, ST identified Galectin-3–positive macrophages, *GPNMB*-expressing regenerative macrophages, and osteopontin/TGF-β signaling circuits as drivers of fibrosis or repair, offering translational insights into shared sarcopenia pathology. Collectively, these spatially resolved findings highlight ECM remodeling and fiber-type–specific stress pathways as common denominators in sarcopenia progression.

The current lack of large-scale human spatial transcriptomic datasets represents a critical gap in sarcopenia research. Comprehensive ST mapping of aged skeletal muscle, including variation across anatomical sites, levels of physical activity, and comorbid conditions, will be essential for validating preclinical findings, refining therapeutic targets, and accelerating translational progress. To advance this next phase of research, coordinated consortia-based efforts and standardized tissue-processing pipelines will be crucial.

Future research integrating ST with complementary multi-omics technologies, such as single-cell sequencing, proteomics, and metabolomics, could further unravel the mechanisms underlying sarcopenia [116,117]. Such integration holds the potential to identify novel therapeutic targets and support the development of tailored interventions. In addition, the application of ST to transcriptome-based drug repositioning strategies opens up new avenues for therapeutic innovation [118,119]. By providing spatially resolved molecular insights, ST can refine the identification of druggable targets and pathways within specific cellular niches of aging muscles. This could accelerate the discovery of existing drugs that can be repurposed to counteract sarcopenia, offering a cost-effective and efficient path toward therapeutic development.

## CRediT author statement

**Byeong-Don Min:** Writing - Original Draft. **Chae Young Hwang:** Writing - Original Draft. **Doyeong Kim:** Writing - Original Draft. **Sang-Yun Kim:** Visualization. **Jayasinghage Nirmani Chathurangika Jayasinghe:** Writing - Review & Editing. **Minh Nhat Tran:** Writing - Review & Editing. **Sang-Min Park:** Conceptualization, Writing - Original Draft. **Ki-Sun Kwon:** Conceptualization, Writing - Review and Editing, Project administration.

## Conflicts of interest

Ki-Sun Kwon serves as CEO of Aventi Inc. Chae Young Hwang serves as CTO of Aventi Inc. The other authors declare no competing interests.
